# Black Seed *(Nigella Sativa)* and its Constituent Thymoquinone as an Antidote or a Protective Agent Against Natural or Chemical Toxicities

**Published:** 2017

**Authors:** Alireza Tavakkoli, Ali Ahmadi, Bibi Marjan Razavi, Hossein Hosseinzadeh

**Affiliations:** a *Department * *of * *Pharmacodynamy * *and * *Toxicology, School * *of * *Pharmacy, * *Mashhad University of Medical Sciences, Mashhad, Iran. *; b *Targeted Drug Delivery Research Center, Department of Pharmacodynamy and Toxicology, School of Pharmacy, Mashhad University of Medical * *Sciences, * *Mashhad, Iran.*; c *Pharmaceutical Research * *Center, * *Department * *of Pharmacodynamics and Toxicology, School of Pharmacy, Mashhad University of Medical Sciences, Mashhad, Iran.*

**Keywords:** *Nigella sativa*, Thymoquinone, Antidote, Protective, Natural toxin, Chemical toxin

## Abstract

*Nigella sativa* *(N. sativa),* which belongs to the botanical family of Ranunculaceae, is a widely used medicinal plant all over the world. *N. sativa* seeds and oil have been used in the treatment of different diseases. Various studies on *N. sativa* have been carried out and a broad spectrum of its pharmacological actions have been established which include antioxidant, antidiabetic, anticancer, antitussive, immunomodulator, analgesic, antimicrobial, anti-inflammatory, spasmolytic, and bronchodilator. This is also indicated that the majority of the therapeutic effects of *N. sativa* are due to the presence of thymoquinone (TQ) that is the main bioactive constituent of the essential oil. According to several lines of evidence, the protective effects of this plant and its main constituent in different tissues including brain, heart, liver, kidney, and lung have been proved against some toxic agents either natural or chemical toxins in animal studies. In this review article, several *in-vitro* and animal studies in scientific databases which investigate the antidotal and protective effects of *N. sativa* and its main constituents against natural and chemical induced toxicities are introduced. Because human reports are rare, further studies are required to determine the efficacy of this plant as an antidote or protective agent in human intoxication.

## Introduction

**Figure 1:**
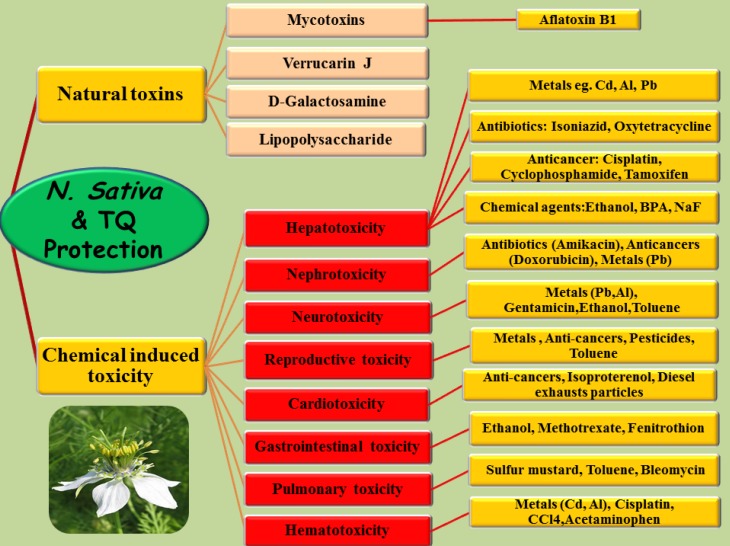
Schematic description of *N. sativa* and TQ against toxicities induced by natural toxins and chemicals in different tissues

*Nigella sativa*, which belongs to the botanical family of Ranunculaceae, commonly known as black seed. It grows in Eastern Europe, the Middle East, and Western Asia (1). *N. sativa* seeds and oil have been extensively used in treatment of different diseases throughout the world. *N. sativa *is included in the list of natural drugs in different medicines including Tibb-e-Nabavi (The medicine of Prophet Mohammad), Unani Tebb, and Indian traditional medicine (2). In traditional remedy, *N. sativa* seeds are commonly used as a spice and carminative (1). In addition, several properties such as liver tonics, diuretics, digestive, anti-diarrheal, appetite stimulant, analgesics, and anti-bacterial have been attributed to this plant (1-3). *N. sativa* has been investigated for its biological effects and therapeutic potential and shown to have broad spectrum of activities including antidiabetic (4, 5), anticancer (6), immunomodulator (7), analgesic (8), antimicrobial (9), anti-inflammatory (8), spasmolytic (10), bronchodilator (11), hepato-protective (12, 13), renal protective (14-16), gastro-protective (17), and antioxidant (18) properties. *N. sativa* seed contains more than 30% fixed oil and 0.4 to 0.45% volatile oil (19). Thymoquinone (TQ) is an abundant component of black seed oil extract (20). TQ has a strong antioxidant potential due to its free radicals scavenging activity (18). There are many reports on its biological activities including analgesic, anti-inflammatory (8, 21), anti-tussive (22), anti-hypertensive (23), anti-diabetic (24), anti-bacterial (25), and anticancer (26). In addition, the protective effects of *N. sativa* and its main constituents in different tissues including brain (27- 29), heart (30), liver (12), kidney (14), lung (31, 32), etc have been established against some toxic agents. In this review article, several *in-vitro* and animal studies in scientific databases which investigate the antidotal and protective effects of *N. sativa* and its main constituents against natural and chemical induced toxicities are introduced (Table 1, Figure 1). Because human reports are rare, further studies are required to determine the efficacy of this plant as an antidote or protective agent in human intoxication.

*Natural toxins*

According to documents, *N. sativa* and its main component (TQ) exhibit antidotal effects against some natural toxins including mycotoxins (33, 34), D-galactosamine (35), and lipopolysaccharides (LPS) (36). These effects might be due to their antioxidant (33), improvement in antioxidant defense system (37), improvement in disturbed biochemical injury markers (35), and antiapoptotic (38) effects. 

*Mycotoxins*

*Aflatoxin B1 (AFB1)*

AFB1 is an aflatoxin produced by *Aspergillus flavus* and *A. parasiticus* (39).  TQ showed a protective effect against AFB1-induced hepatotoxicity in mice by reduction of hepatic injury markers including AST (aspartate aminotransferase), ALT (alanine aminoteransferase), and ALP (alkaline phosphatase) and also via preventing necrosis and degradation of hepatic tissue. Malondealdehyde (MDA), which is an indicator of lipid peroxidation was markedly increased in AFB1-toxicated mice in liver, whereas TQ pretreatment significantly prevented MDA production. Histopathological effects such as inflammation, necrosis, disruption of hepatocytes, hyperplasia of kupffer cells, infiltration of mononuclear cells and increased diameter of hepatocytes were observed as signs of toxicity. TQ reduced the number of inflammatory cells and ameliorated the histopathological changes (33). Another study revealed that aflatoxin-contaminated diet induced haematological and biochemical changes in rats. *N. sativa* and *Syzygium aromaticum* oil significantly restored theses changes. The protective effect of *N. sativa* oil was more than *Syzygium aromaticum* oil (34). 

*Verrucarin J*

Verrucarin J is one of the most important trichothecene which is produced by several genus of fungi. Verrucarin J can contaminate foods of both humans and animals (40). Male rats treated with a sublethal dose of verrucarin J showed an increase in blood and liver tissue levels of thiobarbituric acid reactive substances (TBARs), superoxide dismutase (SOD) and 5-nucleotidase. These effects were decreased largely by *N. sativa*. Similarly, the decrease in levels of glucose and zinc in blood as well as reduced GSH and glucose-6-phosphate dehydrogenase in liver tissue by verrucarin J was improved by *N. sativa *(37).

*D-galactosamine*

D-galactosamine is an amino sugar which can lead to haptic damage and necrosis just after a single dose administration. Lipopolysaccharide (LPS) sensitizes liver against multiple toxins including galactosamine (41). A synergistic action of LPS and galactosamine can induce fulminant hepatitis. TQ maintained AST, ALT, and ALP levels near to normal in D- galactosamine /LPS-intoxicated rats. Furthermore, TQ improved degeneration of liver architecture, infiltration, and inflammation in hepatic tissues (35).

*Lipopolysaccharide (LPS)*

 Lipopolysaccharide (LPS;endotoxin) is a component of gram-negative bacteria which exhibits a potent inflammatory response in mammals (42). Antioxidant and antiapoptotic effects of *N. sativa* against endotoxemia in rats were manifested by normalization of reduced liver GSH and reversing the increase in the level of MDA and the activity of caspase-3 enzyme in the liver. It also reduced the activities of serum tumor necrosis factor-alpha (TNF-α) and bilirubin levels as well as activities of ALP, gamma-glutamyl transferase (γ-GT), and ALT (38).

*Chemical induced toxicity*

*Protective effects of black seed against chemical induced hepatotoxicity*

*Metals*

*Cadmium (Cd)*

Cadmium (Cd^2+^) is a toxic heavy metal and its major target is liver (42). Exposed mice to CdCl_2_ showed a significant elevation in SOD activity and a marked decline in catalase (CAT) activity which were both prevented by pretreatment with TQ. The levels of protein carbonyl, which is an oxidant marker, increased remarkably in case of CdCl_2_ pretreatment but declined considerably if it was combined with TQ (43).

*Aluminum (Al)*

Aluminum (Al) is the third most abundant element in nature (44). Aluminum salts are widely used in drinking water for purification purposes which allows its easy access into the body via gastrointestinal tract and lung tissue (45, 46). Oxidative stress was observed in rats exposed to Al, manifested by an increase in hepatic MDA levels, a decline in GSH and a fall in glutathione peroxidase (GPx), SOD and CAT activities (47, 48). Administration of *N. sativa* oil alleviated the lipid peroxidation in liver and reduced MDA levels significantly, as well as modulated the disturbed activities of hepatic antioxidant enzymes (49).

*Lead (Pb)*

Lead, which plays a major role in today’s industry, is known for its toxic effects on multiple organs including kidney and Liver (50). TQ is proved to alleviate lead acetate toxicity in rats. In terms of biochemical parameters, co-treatment with TQ prevented the serum AST from rising and provided no significant change in serum total protein and albumin, the opposite of what was seen in rats fed only on lead acetate. Periportal necrosis of hepatocytes and inflammatory infiltration were observed in lead acetate-treated rats, whereas TQ maintained the normal structure of liver (51).

*Antibiotics*

*Isoniazid*

Isoniazid (INH) is a first-line medication in prevention and treatment of tuberculosis. One of its main side effects is mild to severe hepatotoxicity (52, 53). INH-induced hepatotoxicity in rats was manifested by sinusoidal dilatation (mainly lobular), hepatocellular necrosis, moderate portal inflammation and degeneration of hepatocytes. These inflammatory signs were not observed in rabbits pretreated with *N. sativa*. In terms of hepatic biomarkers, *N. sativa* also significantly reversed the elevated levels of serum ALT, AST, ALP, and bilirubin caused by INH (54).

*Oxytetracyclin*

Oxytetracyclin is a widely used antibiotic for treating bacterial infections caused by gram-negative and gram-positive microorganisms (55). Oxytetracyclin, however, can have serious side effects like hepato-renal toxicity in high doses (56, 57). Oral administration of oxytetracyclin in rabbits elevated ALT and AST more than two folds of the normal level. It also increased ALP, lactate dehyrogenase (LDH), cholesterol ,and total serum bilirubin. Moreover, renal biomarkers like serum urea, uric acid and creatinine were increased. *N. sativa *oil and ascorbic acid (in combination or individually) modulated the rises in liver and kidney injury markers. In terms of lipid peroxidation and antioxidant capacity in both organs, treatment with oxytetracyclin remarkably increased MDA and reduced the CAT, SOD and glutathione (GSH) activities. Combination of *N. sativa *oil and ascorbic acid restored the antioxidant status and MDA levels back to normal and showed greater therapeutic effect than when each of them was used alone (58).

*Anti-cancers*

*Cisplatin*

Cisplatin is a well-known anticancer drug prescribed mainly to treat solid tumors. It has to be used cautiously due to its toxic effects on liver and kidney (59). The main mechanism of cisplatin hepatotoxicity, which is indicated by a fall in GSH levels and a rise in MDA levels, is through oxidative stress (60). Cisplatin can induce generation of reactive oxygen specious (ROS) which in turn could lead to DNA damage and membrane lipid peroxidation (61). Treatment of cisplatin-intoxicated rats with TQ showed a significant improvement in the elevated levels of some hepatic biomarkers and manifested a marked increase in GPx activity. Enhancement in activities of antioxidant enzymes such as SOD and CAT was observed which led to a great reduction in lipid peroxidation (62).

*Methotrexate*

Methotrexate (MTX) is a chemotherapeutic agent which can cause hepatotoxicity as a side effect (63). Administration of *N. sativa* oil in rats intoxicated with MTX manifested less degeneration in hepatic tissues through anti-oxidant activity (64). Another study was conducted with the aim of assessing the antidotal effect of black cumin on children diagnosed with acute lymphoblastic leukemia who were under MTX treatment. It was suggested that black cumin significantly recovered the increased levels of serum bilirubin, serum ALT, AST and ALP but did not have a remarkable effect on serum total protein, serum albumin, and globulin (65). El-Sheikh *et al* reported that using TQ concurrently with MTX reversed up regulation of iNOS (inducible nitric oxide synthase), necrosis factor-𝜅B, cyclooxygenase-2, and caspase 3 in rat liver which respectively demonstrates anti-oxidant, anti-inflammatory, and anti-apoptotic effect of TQ (66).

*Cyclophosphamide (CTX)*

Cyclophosphamide (CTX) is an alkylating agent with significant therapeutic importance in a wide range of conditions including cancer (67). However, it could induce overproduction of reactive oxygen species which accounts for its systemic toxicity (68). CTX treatment resulted in a considerable increase in serum ALT and AST in rats. Administration of *N. sativa* oil and TQ after and before CTX induced recovery of both enzyme activities. The same effect was observed in levels of ALP and creatine phosphokinase (CPK) in case of pretreatment with *N. sativa* oil or TQ (69).

*Tamoxifen*

Tamoxifen is an antistrogen used in breast cancer therapy and also as a chemopreventive agent in health woman with high risk of developing breast cancer (70, 71). Tamoxifen is a potent hepatocarcinogen with tumor-initiating and tumor-promoting properties due to its excessive oxygen radical production (72-75). Serum liver enzymes, AST, ALT, ALP, LDH, γGT, and total bilirubin were significantly elevated in tamoxifen -treated rats. These changes were reduced by administration of TQ. Levels of SOD and GSH were increased and the elevated lipid peroxidation and TNF-α were attenuated upon administration of TQ to tamoxifen -intoxicated rats (76).

*Imidacloprid*

Imidacloprid, is a member of the neonicotinoid insecticide class which acts on the nervous system by blocking postsynaptic acetylcholine receptors and is highly effective against a variety of insects (77).

A decline in the levels of serum ALT, AST, ALP, and MDA in rats exposed to imidacloprid was an indication that TQ is an useful agent in reducing the oxidative stress caused by exposure to this insecticide (78).

*Carbon Tetrachloride (CCl4)*

Carbon tetrachloride (CC14) is a xenobiotic which produces hepatotoxicity in humans as

well as in animals (79, 80). A single dose of CC1_4_ induced hepatotoxicity, manifested biochemically by significant elevation of serum enzyme activities, such as ALT , AST and LDH in mice. These levels fell sharply after treatment with TQ. TQ raised hepatic catalase activity as well (81). In another study conducted on Wistar rats, administration of CCl4 caused a rise in lipidperoxidase levels of liver and kidney and reduced GSH in these two organs markedly. Moreover, in terms of histopathological observations, it produced fatty degeneration, distended hepatocytes and distortion of hepatic architecture. Treatment with *N. sativa* (750mg/Kg) had the best effect on restoring the antioxidant capacity back to normal and protected the hepatic lesions caused by CCl4 (82).

*Tert-butyl hydroproxide (TBHP)*

Tert-butyl hydroproxide is a hepatotoxic agent which in case of exposure stimulates rapid oxidation of intracellular GSH, and pyridine nucleotides, as well as leakage of cytosolic enzymes (83, 84).

Administration of TQ increased the reduced viability of TBHP-treated rat hepatocytes besides lessening the leakage of AST and ALT (85).

*Ethanol*

Ethanol is a well-known hepatotoxic agent which caused substantial increases in ALT and AST levels after application in rats. It also elevated triglyceride and MDA levels notably. Treatment with *N. sativa* oil provided a significant drop in increased transaminase levels and reduced triglyceride and MDA (86, 87).

*Sodium Fluoride (NaF)*

Fluoride anion is an agent which contributes to dental protection and prevents osteoporosis in small doses, but in case of excessive exposure it can interfere with metabolic pathways involving lipids, carbohydrates and proteins (88, 89). The consumption of NaF in rats significantly disrupted the antioxidant functionality of liver by reducing SOD, CAT and GSH levels. The elevated levels of AST, ALT, ALP and LDH as well as a remarkable decrease in serum protein profile were also observed. Administration of TQ with NaF restored the antioxidant activity of liver and reversed the decline in serum protein levels (90).

*Bisphenol A (BPA)*

Bisphenol A (BPA) is a compound widely used in plastic and resin industry and is a main component of plastic baby bottles, as well as food and beverage containers (91). BPA has an extended range of adverse effects and can cause oxidative stress in liver (92). Reduction in hepatocytes viability and their mitochondrial function were reported in rats treated with BPA (93). Administration of TQ greatly normalized suppressed enzymatic and non-enzymatic antioxidants such as GSH, GPx, GST, SOD, and CAT. It also reduced elevated levels of hepatic biomarkers and decreased lipid peroxidation (94).

*Acetaminophen*

Severe hepatotoxicity caused by acetaminophen overdose is well documented (95). It also leads to a significant increase in serum ALT and total nitrate/nitrite, hepatic lipid peroxides, and depletion of hepatic GSH and ATP (96). Acetaminophen overdose can result in liver necrosis and be fatal (97). TQ produced a marked normalization of the acetaminophen-induced increase in serum nitrate/nitrite and hepatic ALT. A substantial increase in lowered ATP and GSH levels was manifested (98). It was also found that co-administration of TQ with acetaminophen significantly elevated lowered glutathione peroxides (99). Furthermore *N. sativa* extract caused resumption of liver architecture and lessened tissue necrosis and infiltration (100).

*Sodium valproate*

Sodium valproate is a widely used drug in the treatment of epilepsy while it is known for its hepatotoxic effects which is characterized by elevated serum AST and ALT as well as a fall in non-protein sulfhydryls and an increase in lipid peroxidation in hepatic cells. Co-treatment of sodium valproate and TQ ameliorated the raised AST and ALT levels but did not prevent increased lipid peroxidation. Moreover, the co-treatment improved the reduction of hepatic glutathione induced by sodium valproate (101).

*Indomethacin*

Indomethacin is a NSAID used in the treatment of inflammatory disorders. In high doses however, it can induce hepatotoxicity and hepatocyte death by activating multiple stress pathways. TQ is proved to have oxidant- scavenging abilities and the potential to decrease apoptotic changes which can serve as a protective mechanism against indomethacin -induced toxicity (102).

*Antiretroviral drugs*

HAART (Highly Active Antiretroviral Therapy) is the use of a combination of drugs such as Lamivudine, Zidovudine, and Efavirenz to control retroviral infections which can lead to hepatotoxicity (103). In HAART treated rats, the hepatic biomarker levels such as AST, ALT, ALP, and GGT were significantly increased and albumin concentration dropped sharply. Besides, there were signs of histopathological damage in liver tissues. Administration of *N. sativa* restored the biochemical and histopathological changes to normal through its antioxidant activity (104).

*Protective effects of black seed against chemical induced nephro- or uro-toxicity*

*Antibiotics*

*Amikacin*

Amikacin is an aminoglycoside in severe infections. However, its use is associated with undesirable renal toxicity (105). In renal tissue of amikacin-treated rats, MDA level elevated and GSH content reduced which indicates oxidative stress. In addition, creatinine, urea and uric acid levels increased which indicates renal dysfunction. Histopathological examination showed severe vacuolation of renal tubular cells, massive tubular necrosis, renal cast and mononuclear cell infiltration. In rats received N*. sativa* oil with amikacin, all of mentioned markers significantly declined to normal status (105). 

*Gentamicin*

Gentamicin is effective against gram negative bacterial infection in human and animals (106). Nephrotoxicity is a main complication of its therapeutic doses. Renal failure occurs in about10–30% of patients receiving the drug (107). The results of some studies showed that gentamicin caused moderate proximal tubular damage, glomerular and tubular necrosis, interstitial nephritis and desquamation of the tubular epithelial cells in rat's renal cortex (106, 107). It also significantly increased the levels of creatinine and urea, and decreased the levels of total antioxidant status and GSH in kidney cortex. Treatment with *N. sativa* oil enhanced rat's growth, and produced a dose-dependent amelioration of the biochemical and histological indices of gentamicin nephrotoxicity that was statistically significant at doses of 1.0 and 2.0 ml/kg/day for 10 days (106).

In another study, in comparison with gentamicin group, *N. sativa* administration caused significant decreases in MDA level and nitric oxide generation and increases in SOD and GSH-Px activities. So, *N. sativa* ameliorates gentamicin-induced oxidative stress (108). Moreover, the synergistic nephroprotective effects of vitamin C and *N. sativa* oil have been shown. Combination of vitamin C and *N. sativa* oil decreased the levels of nephrotoxicity markers such as serum creatinine, BUN, and antioxidant activity as compared with gentamicin treated rabbits (109). It is also reported that TQ supplementation completely reversed the decrease in ATP and the ATP: ADP ratio induced by gentamicin. Therefore, TQ prevents the energy decline in kidney tissues and so protected gentamicin-induced renal failure (110).

Sayed-Ahmed and Nagi (2007) reported that TQ supplementation resulted in a complete reversal of the gentamicin-induced increase in BUN, creatinine, thiobarbituric acid reactive substances and total nitrate/nitrite and decrease in GSH, GPx, CAT, and ATP to control values. So, TQ supplementation can prevent the development of gentamicin-induced acute renal failure (107).

*Oxytetracycline*

Oxytetracyclin is a widely used antibiotic for treating bacterial infections caused by gram-negative and gram-positive microorganisms (55). Oxytetracyclin, however, can have serious side effects like hepato-renal toxicity in high doses (56, 57). Based on the evidences from animal studies, *N. sativa* oil co-administration could alleviate oxytetracycline -induced increase in serum biochemical renal injury markers and lipid peroxidation. Moreover, *N. sativa* oil could improve decrease in tissue antioxidant biomarkers (58).

*Vancomycin*

Vancomycin is a kind of glycopeptide antibiotic with antibacterial effect against aerobic and anaerobic gram-positive bacteria. It was reported that levels of serum blood urea nitrogen (BUN), creatinine (Cr) and kidney tissue MDA were increased in the rats treated by vancomycin whereas TQ administration significantly lowered them (111).

*Anti-cancers*

*Doxorubicin*

Doxorubicin is an antitumor drug that like other chemotherapy drugs has many side effects, the most dominant of which is severe nephrotoxicity. In the renal tissue of doxorubicin -treated rats the activities of SOD and GST were significantly decreased and lipid peroxidation increased. In addition, doxorubicin caused significant changes in renal levels of inflammatory mediators: increase in TNF-a and IL-6 levels and marked decrease in IL-10 levels. Remarkably TQ restores all mentioned markers toward normal values (112).

Badary *et al* (2000) reported that treatment with TQ significantly suppressed doxorubicin -induced proteinuria and lowered the levels of triglycerides, total cholesterol, and lipid peroxides in the rat kidneys (113).

*Cisplatin*

Cisplatin, is a chemotherapeutic drug which is widely used for treatment of several kinds of human disease. Administration of cisplatin is a common cause of acute renal failure, which is a life-threatening illness that continues to have a high mortality. Cisplatin-induced renal injury is associated with an elevation in protein levels of the efflux transporters MRP2 and MRP4 while expression of organic anion and cation transporters (OATs and OCTs) was reduced. Co-administration of TQ with cisplatin reversed down-regulation of OAT1, OAT3, OCT1, and OCT2 as well as up-regulation of MRP2 and MRP4. It was also proved that TQ can alleviate cisplatin-stimulated oxidative stress makers, lipid peroxidation status, and other nephrotoxicity markers (114). TQ treatment also significantly decreased cisplatin-induced elevated serum urea and creatinine levels (115). However, Hadjzadeh *et al* reported that use of *N. sativa* seeds had little effects on biochemical parameters but relatively recovered histopathologic properties of the kidneys (116). One study was performed in order to show the renal toxicity of cisplatin and the way ARF (acute renal failure) was caused by this anticancer drug and eventually to measure the ameliorative effect of TQ on this toxicity. Cisplatin can aggravate the pulmonary dysfunction produced by diesel exhaust particles (DEP). Cisplatin alone, induced significant rises in urea and neutrophil gelatinase-associated lipocalin (NGAL) and remarkably reduced creatinine clearance. DEP alone did not manifest a marked effect on renal parameters and did not significantly change the aforementioned actions of cisplatin. TQ proved to be useful in restoring the normal levels of the indices especially urea. Tubular necrosis and dilation were observed in cisplatin-treated rats and the conditions were exacerbated in case of co-treatment with DEP. The damage was lessened by treatment with TQ (117).

*Ifosfamide*

Ifosfamide is a synthetic structural isomer of cyclophosphamide that has been approved for concurrent use with other drugs in the treatment of metastatic germ-cell testicular cancer and some sarcomas. Nephrotoxicity is a well-known complication of ifosfamide therapy. Renal fanconi syndrome is one of renal toxicities of ifosfamide and is characterized by a generalized disorder in proximal tubule transport (118). It was proved that TQ provides protection against ifosfamide -induced fanconi syndrome in rats as well as improving its anti-tumor effect in mice (119).

*Methotrexate (MTX)*

MTX is one of the most widely used anticancer drugs. One of the most prominent toxicities caused by MTX chemotherapy is nephrotoxicity (66). It is revealed that co-administration of TQ with methotrexate could reverse MTX-induced oxidative and nitrosative stress, as well as inflammatory and apoptotic signs in rats kidneys. TQ also ameliorated kidney dysfunction and histological damages caused by MTX (66).

*Bromobenzene*

Bromobenzene is a solvent in the chemical industry and chemical intermediates. Bromobenzene metabolites are highly hepatotoxic while secondary metabolites are highly nephrotoxic which are conjugated to bromoquinones that can accumulate in the kidney and are nephrotoxic. The observed low level of serum protein and elevated levels of BUN and creatinine in bromobenzene**-** treated rats indicate renal dysfunction. Administration of *N.sativa* oil returned BUN and creatinine levels to normal level and significantly increased the reduced serum protein level (120).

*Nitrilotriacetate Fe*

Nitrilotriacetic acid is a constituent of various domestic and hospital detergents and is a common water contaminant (121). The iron complex of nitrilotriacetic acid (Fe-NTA) is nephrotoxic. A study by Khan and Sultana (2005) demonstrated *N. sativa* could suppress hyperproliferative response and reduce tumor promotion in kidney tissue of rats (122).

*Cyclosporine A*

Nephrotoxicity is the main secondary effect of cyclosporine A treatment which is used for the prevention of allograft rejection in solid organ transplantation. Uz *et al* (2008) reported that co-administration of *N. sativa* oil with cyclosporine A ameliorate deterioration in the renal function and morphology was caused by cyclosporine A. It also attenuated the oxidative stress induced by cyclosporine A (123).

*Metals*

*Lead (Pb)*

Lead caused significant elevations in AST, urea, creatinine, total cholesterol, and triglycerides in serum as well as significant decrease in serum total protein and albumin. Co-administration of *N. sativa* seeds with lead acetate improved the biochemical parameters and reduced the damaged areas in the liver and kidneys (58).

*Mercuric chloride*

Mercuric chloride (HgCl2) is a potent nephrotoxicant that has been widely used in animal models of acute renal failure. Fouda *et al* (2008) reported that in rats who received a nephrotoxic dose of HgCl2, changes in the renal MDA, GSH content, GPx, and CAT activities were observed. This rapid increment of the biomarkers of oxidative stress was associated with marked renal cellular injury. In addition, some signs of histological damage, apoptotic events, and proliferative reactions were seen. The deterioration of antioxidant enzymes and histological damage caused by HgCl_2_were markedly improved by TQ treatment . Apoptosis and proliferative reactions were also reduced, so TQ may be clinically useful in inorganic mercury intoxication (124).

*Acetaminophen*

Renal insufficiency occurs in approximately 1-2% of patients with acetaminophen overdose (125). Aycan *et al* (2015) reported that in the TQ-treated group, urea and creatinine levels were lower than the group received acetaminophen whereas MDA and Nitric oxide levels were higher. The results showed that IP. administration of TQ caused significant improvement in biochemical, antioxidant ,and histopathological changes induced by acetaminophen in rats (126). Another study revealed that co-administration of silymarin and TQ had a better ameliorative effect in case of acetaminophen toxicity and reduced urea and creatinine levels sufficiently more than when each of them was used alone (127).

*Protective effects of black seed against chemical induced neurotoxicity*

*Metals*

*Lead (Pb)*

Treatment with lead causes wide spectrum of histopathological brain damages in rats includings degeneration of endothelial lining of brain blood vessels with perivascular cuffing of mononuclear cells consistent to lymphocytes, congestion of choroid plexus blood vessels, ischemic brain infarction, chromatolysis and neuronal degeneration, microglial reaction and neuronophagia, degeneration of hippocampal and cerebellar neurons and axonal demyelination. However, TQ supplement with lead acetate markedly decreased the incidence of those pathological lesions (128).

*Aluminum (Al)*

Al is toxic to the central nervous system, Kamal and Kamal (2013) reported that administration of Al in the rat cerebellum caused a significant reduction in the number of Purkinje cells and induced some damages such as cytoplasmic vacuolation, dilatation of Golgi cisternae, and mitochondria with dilated cristae in Purkinje cells. Granule cells showed mitochondria with destroyed cristae. The immune reaction for caspase-3 was intense compared with that of the control group. Co-administration of *N.sativa* oil remarkably protected the neurons against these changes (129).

*Gentamicin*

Nephrotoxicity and ototoxicity are the most common problems during treatment with aminoglycosides such as gentamicin. Auditory brainstem response - thresholds significantly increased in gentamycin received rats. Sagit *et al*. (2014) showed that the mean auditory brainstem response values and numbers of apoptotic cells did not significantly increase in the group receiving gentamicin plus TQ compared to those receiving gentamicin alone. TQ (20 mg/kg) has protective effects on gentamicin-induced ototoxicity (130).

*Ethanol*

Exposure to ethanol during early development triggers severe neuronal death by activating multiple stress pathways and causes neurological disorders, such as fetal alcohol effects or fetal alcohol syndrome. Ullah *et al*. (2012) reported that ethanol exposure in rat prenatal cortical neurons induces elevation of cytosolic free calcium [Ca^2+^] and decreases normal physiological mitochondrial transmembrane potential (ΔψM) which both of them returned to normal status by TQ. Increased [Ca^2+^] and decreased ΔψM significantly reduced the expression of a key anti-apoptotic protein (Bcl-2), increased expression of Bax, and stimulated the release of cytochrome-c from mitochondria. TQ also inhibited the apoptotic cascade by increasing Bcl-2 expression (131).

*Toluene*

Toluene is an industrial organic solvent with extensive uses. It is revealed that toluene chronic exposure caused severe degenerative changes in rat’s frontal cortex neurons; such as shrunken cytoplasma, severely dilated cisternae of endoplasmic reticulum, markedly swollen mitochondria with degenerated cristae and nuclear membrane breakdown with chromatin disorganization. TQ treatment markedly prevented mentioned degenerative changes, although moderately mitochondrial swollen was still observed in neurons with TQ treatment (132).

*Propoxur*

Propoxur is a carbamate insecticide with wide uses especially for household pests. It induces lipid and protein peroxidation and decreases acetylcholine esterase activity in cerebellum, cortex, and hippocampus. It is also revealed that propoxur significantly reduced the enzymatic antioxidant (SOD, CAT, GSH-Px and GST) activities and non-enzymatic antioxidant (GSH) levels. However, treatment with *N. sativa* oil can ameliorate the propoxur-induced toxicity and oxidative stress in the three brain regions (133).

*Protective effects of black seed against chemical induced reproductive toxicity*

*Metals*

*Cadmium (Cd)*

Sayed *et al*. (2014) reported that Cd long-term exposure caused oxidative stress, spermatological damage, histopathological alterations, and decrease in serum testosterone level in testes, epididymis, and accessory glands in male rats. On the other hand, co-administration of TQ and Cd ameliorated all the damages. Remarkably, no significant difference was observed between Cd-treated and TQ-treated rats in sperm count (134). Another study demonstrated that Cd acute exposure induced oxidative stress indicated by decrease in antioxidants and increase in oxidative enzymes. Cd also elevated the expression of iNOS, TNF-α, COX-2, NF-κB, and caspase-3 in seminiferous tubules cells. TQ treatment alleviated these changes and markedly ameliorated the cadmium-induced damage of testicular tissue and preserved spermatogenesis in most of seminiferous tubules (135).

*Lead (Pb)*

A study by Mabrouk and Ben Cheikh (2015) demonstrated that TQ co-treatment could improve lead induced testicular oxidative stress in rats by decrease in the elevated levels of oxidative enzymes and increase in the level of glutathione significantly (136).

*Anti-cancers*

*Methotrexate*

It was revealed that mice received methotrexate resulting in interstitial space dilatation, edema, vasculitis, abnormalities of Leydig cells, severe disruption of the seminiferous epithelium, and reduced diameter of the seminiferous tubules; however, TQ co-treatment significantly reversed these histopathological changes and prevented methotrexate-induced increase in the myeloperoxidase activity, but the levels of MDA did not differ among the groups (137).

*Cisplatin*

Awadalla (2012) reported that cisplatin negatively affected on MDA level as well as histological structure of the testes and *N. sativa* oil restored these changes to those of control (138).

*Cyclophosphamide*

Cyclophosphamide is one of the most harmful alkylating agents. It can cause oxidative stress due to the over-production of ROS. It affects the DNA of replicating cells and rapidly multiplying cells especially in the gonads and pituitary which results in miscoding, cross-linking, and DNA breakage (139). A prophylactic effect of *N. sativa* in the reproductive system of female mice has been shown. In mouse ovarian tissue exposed to *N. sativa* oil before cyclophosphamide exposure significant protection on the fine structure of follicles was observed. Furthermore, the survival rates of normal follicles in group received *N. sativa* oil were higher than cyclophosphamide-treated group (140).

TQ administration significantly alleviated the percentage of defects blastomeres of type c and embryo fragmentation grade IV in cyclophosphamide-treated mice; so, it could be a suitable supplementation for preserving fair-quality embryos and achieving full term pregnancy (139). 

Cyclophosphamide treated mice induced reduction of testes weights, epididymides and sperm count with high percentage of acrosome reacted sperm possibly to spontaneous acrosomal reaction caused by free radicals. Co-administration of *N. sativa* ethanol extract and cyclophosphamide elevated sperm count but had no effect on their histology (141).

*Pesticides*

*Acetamiprid*

Acetamiprid is an odorless neonicotinoid insecticide (142). Acetamiprid caused reproductive toxicity in male rats indicated by a decrease in body weight gain, relative weights of reproductive organs (testis, epididymis and seminal vesicle), spermatids number, sperm count, sperm motility, and testosterone levels. It also induced histopathological changes including tubular atrophy, disorganization, and degenerative aspect of the seminal epithelium in some seminiferous tubules marked by spermatogenesis perturbation and poor sperm and lso presence of sloughing cell debris in their lumens. It is revealed that *N. sativa* oil co-administration modulated these acetamiprid-induced reproductive adverse effects (143).

*Chlorpyrifos*

 Chlorpyrifos is a pesticide that can induce toxic effects on male rat reproductive system indicated by a decrease in sperm count and production, sexual hormones, body weight, and relative weight of reproductive organs accompanied by increase in dead and abnormal sperms. Chlorpyrifos treatment also causes oxidative stress. Coadministration of *N. sativa* oil and chlorpyrifos ameliorated chlorpyrifos toxic effects (144).

*Toluene*

Toluene is a volatile organic compound widely used as an industrial solvent. It can also be found as an air pollutant in homes and buildings. Exposure to high levels of toluene inhalation is known to induce reproductive and developmental toxicity. Kanter (2011) reported that TQ co-administration markedly lowered the reactivity and the number of germ cell apoptosis and improved cell organelles damages in toluene-treated rat's testis. The results showed that chronic toluene exposure decreased spermatogenesis and mean seminiferous tubule diameter. TQ improved histopatological damages induced by toluene in rats (145).

*Colchicine*

Colchicine is one of the gout therapy drugs that have a narrow therapeutic index and its toxicity is associated with high mortality rate (146). Colchicine caused histopathological damages in rat testes as well as decrease in spermatogenesis and testosterone plasma level and low positive reaction of PAS. *N. sativa* seeds supplementation significantly improved testicular toxicity manifestations induced by colchicine (146).

*Protective effects of black seed against chemical induced cardiotoxicity*

*Anti-cancers*

*Cyclophosphamide*

Cyclophosphamide is an alkylating agent, which is commonly used in most cancer chemotherapy. High therapeutic doses of cyclophosphamide are associated with lethal cardiotoxicity (147). In rat heart tissues, cyclophosphamide causes a significant increase in TBARS and total nitrate/nitrite and a significant decrease in reduced GSH, GPx, CAT, and ATP levels. Interestingly, TQ supplementation completely reversed all cyclophosphamide-induced biochemical changes to their control values (147).

*Doxorubicin*

Doxorubicin is an anthracycline antibiotic which is used as an antitumor agent. It is well known that anthracyclines can induce cardiotoxicity by releasing ROS (148). According to a study by Al-Shabanah *et al* (1998), doxorubicin -induced cardiotoxicity could lead to cardiomyopathy and heart failure. TQ could protect doxorubicin-induced cardiotoxicity as evidenced by significant reduction in creatine phosphokinase and lactate dehydrogenase in rats. This study revealed that TQ could ameliorate doxorubicin cardiotoxicity without decreasing doxorubicin antitumor activity or its plasma and heart levels (148). 

*Isoproterenol*

Isoproterenol induced myocardial injury, is a classical example of excess catecholamines related coronary insufficiency and stress cardiomyopathy. A study revealed that TQ oral administration (dissolved in olive oil) protected the rat’s heart from stress cardiomyopathy induced by isoproterenol. Isoproterenol significantly increased plasma LDH, TBARS, and glutathione reductase , whereas there was a dose related decrease in these markers in TQ treated groups. In addition, TQ reversed decrease in plasma SOD, myocardial GSH/GSSG ratio, and histological changes produced by isoproterenol (149).

*Diesel exhausts particles (DEP)*

Adverse cardiovascular events are most strongly associated with exposure to fine particulate matter (diameter < 2.5 µm). Nemmar *et al* (2011) revealed that TQ pretreatment prevented DEP-induced cardiovascular damages. It is reported that TQ protected DEP-induced decrease of systolic blood pressure, decrease in platelet numbers, leukocytosis, and prothrombotic events. However TQ did not inhibit platelet aggregation *in-vitro* (150).

*Cyclosporine A*

Cyclosporine A is a commonly used immunosuppressive agent in transplant medicine and in the treatment of autoimmune diseases. However, it generates ROS, which causes nephrotoxicity, hepatotoxicity, and cardiotoxicity (151). It is indicated that *N. sativa* oil pre-treatment reduced the subsequent cyclosporine A injury in rat heart, manifested by normalized cardiac histopathology, decrease in lipid peroxidation, improvement in antioxidant enzyme status and cellular protein oxidation (151).

*Methionine*

Homocysteine is a sulfur-containing amino acid which produces during the metabolism of the essential amino acid methionine. Defective metabolism of methionine resulting in hyperhomocysteinemia (HHcy). HHcy is associated with higher risks of coronary, cerebral, and peripheral vascular disease (152). In a study by El-Saleh *et al* (2004), it was showed that methionine-induced hyperhomocysteinemia led to significant increase in the degree of lipid peroxidation and antioxidant enzyme activities (SOD and GPx), decrease in antioxidant status, and elevation in lipid parameters (triglycerides and cholesterol). TQ and black seed oil effectively protected rats against the induction of HHcy due to methionine loading (152).

*Protective effects of black seed against chemical induced gastrointestinal toxicity*

*Ethanol (EtOH)*

The results of a study suggest that TQ could inhibit the development of gastric ulcer caused by ethanol (146, 147). Likewise, TQ protected against the ulcerating effect of alcohol and mitigated most of the biochemical adverse effects induced by alcohol in gastric mucosa, but to a lesser extent than *N. sativa*. *N. sativa* increased gastric GSH content, GST, and enzymatic activities of gastric SOD (154); however, TQ did not statistically change the high SOD activity (153).

*Methotrexate (MTX)*

Labib *et al*. (2009) reported that *N. sativa* oil pretreatment improved MTX-induced diarrhea, food consumption, and body weakness. They also showed that administration of *N. sativa* oil before and after MTX ameliorated gastrointestinal toxicity caused by MTX and maintained mucosal structure (155).

*Fenitrothion*

Fenitrothion is one of the organophosphate insecticides. It is demonstrated that fenitrothion oral administration causes some histopathological changes in salivary glands in rats. Remarkably, administration of natural antioxidants like *N. sativa* oil could be of beneficial effect on prevention of cytotoxicity induced by organophosphate. However, green tea showed more effective results than that of *N. sativa* (156).

*1, 2-dimethyl-hydrazine (DMH)*

DMH is a potent carcinogen that acts as a DNA methylating agent. It is used to induce colon tumors in experimental animals (157). It is revealed that TQ protects and cures DMH-induced initiation phase of colon cancer. It also exerts a protective role at promotion (157).

*Protective effects of black seed against chemical induced pulmonary toxicity*

*Diesel exhaust particles (DEP)*

It was revealed that DEP exposure after 18h caused a significant increase in macrophages and polymorphonuclear cell numbers, total protein, and IL-6 concentrations in mice bronchoalveolar lavage (BAL) fluid, while TQ pretreatment significantly prevented these changes and reduced the number of interstitial inflammatory cells. It also prevented DEP-induced enhancement of airway resistance after increasing concentrations of methacholine (150). TQ also can reverse toxic effects of DEP in combination with cisplatin (117).

*Sulfur mustard*

Sulfur mustard is one of the chemical warfare agents (158). Hossein *et al*. (2008) reported that the tracheal responsiveness to methacholine was significantly higher in sulfur mustard-exposed guinea pigs prevented by *N. sativa* extract co-administration (159). Another study demonstrated that effectiveness of *N. sativa* in decrease of tracheal responsiveness to methacholine was equal to that of dexamethasone and these effects were statistically significant compared to that of sulfur mustard -exposed group (160).

*Toluene*

Toluene administration induced pulmonary toxicity in rats as indicated by severe inflammatory cell infiltration, alveolar obstruction, significant edema, and alveolar hemorrhage, while TQ co-administration significantly improved these changes. TQ also markedly reduced the number of iNOS-positive and apoptotic cells (145).

*Bleomycin*

It was reported that TQ significantly reversed bleomycin-induced depletion of the GSH and elevation in the level of lipid peroxide and the activity of antioxidant enzymes; such as GPx and GST in rat’s lung tissue. The possible way of action is superoxide radical scavengering because TQ is as effective as superoxide dismutase against superoxide (161).

*Protective effects of black seed against chemical induced hematotoxicity*

*Metals*

*Cadmium (Cd)*

Cadmium treatment induces hematotoxicity indicated by decrease in red blood cell (RBC) and white blood cell (WBC) counts, packet cell volume (PCV), hemoglobin (Hb) concentration, and neutrophil percentage . Demir *et al*. reported that *N. sativa* extract increased these lowered markers except WBC count (162). In another study, remarkable membrane destruction and hemolytic changes in Cd-treated rat's erythrocytes were observed, while *N. sativa* co-treatment lessened these dameges and decreased the Cd-induced oxidative stress (163).

*Aluminium (Al)*

A study by Bouasla *et al*. (2014) demonstrated that Al exhibited some hematological damages such as an increase in WBC counts and a marked decrease in erythrocyte counts and Hb content. Moreover, raise of erythrocyte MDA level was associated with a decrease in GSH content, GPS, SOD, and catalase. However, administration of *N. sativa* oil with AlCl3 improved all changes of mentioned parameters (49).

*Cisplatin*

It was revealed that *N. sativa* seed extract protected rats against cisplatin-induced decrease in hemoglobin levels and leucocyte counts (164).

*Carbon tetrachloride (CCl*_4_ *)*

It was reported that CCl_4_ causes significant changes in the haematological parameters and morphological characterizes of peripheral blood cells which included both nucleus and cytoplasm in albino mice. Co-treatment of *N. sativa* aqueous suspension with CCl4 ameliorated these alterations (165).

*Acetaminophen*

It was revealed that decrease in RBC numbers and Hb level as well as increase in WBC numbers and TNF-α level were observed in acetaminophen treated mice. Coadministration of *N. sativa* extract with acetaminophen ameliorated hematotoxicity and immunotoxicity was induced by acetaminophen (166).

## Conclusion

Recently, several natural plants and their active constituents have been used in various studies with the aim to prevent toxicities in different tissues induced by natural or chemical toxicants. The availability, lower price, and less toxic effects of herbal compounds compared with synthetic make them as simple and excellent choice in the prevention of toxicities. This review article summarized different *in-vitro* and *in-vivo* studies in order to find out the role of *N. sativa* and its active constituent, TQ, in prevention against toxicities induced by natural or chemical toxins in different tissues. 

Based on the results of some important investigations, *N. sativa* and its active constituent, TQ, act as an antidote in different intoxications induced by natural toxins including mycotoxins and endotoxins. Some chemicals induced toxicities which have been prevented by *N. sativa* and its active constituent, TQ, include metals (Al, lead, mercury and cadmium), pesticides (imidacloprid, propoxur, acetamiprid, chlorpyrifos, fenitrothion), solvents and detergents (ethanol, CCl4, toluene, nitrilotriacetate) and environmental pollutants (DEP, BPA). Furthermore, *N. sativa* as well as TQ could protect different tissues against some drugs overdose including analgesics, anticancer, immunosuppressive , antibiotics, antiretroviral, and antiseizures. Some mechanisms including antioxidant, anti-inflammatory, free radical scavenging, improvement in the disturbed levels of biochemical markers, modulation of antioxidant defense systems, inhibition of apoptosis and regulatory effects on genes expression , and different signaling pathways are involved in *N. sativa* antidotal effects. According to this review, *N. sativa* has also ability to protect potentially different tissues and organs including liver, kidney, heart, blood, brain, lung, gastrointestinal, and reproductive system against chemical toxins. In conclusion, based on the current review, *N. sativa* has a broad spectrum of protective activities against toxicities induced by either natural or chemical toxicants (Table 1, Figure 1). Since these findings have not yet been established by clinical trials on humans, to verify the antidotal effects of *N. sativa* in human intoxications, human trials should be carried out.

**Table 1:** Protective effects of *N. sativa *or thymoquinone against drugs induced toxicities in different tissues

**Chemical induced toxicity**		**Toxicity**	**Study design, dose and type of ** ***Nigella sativa *** **preparation**	**Protective mechanisms and references**
**Drug family**	**Drug name**			
Antibiotic-aminoglycoside	Gentamicin	Nephrotoxicity	In vivo, rat NSO: (0.2ml/kg and 0.4 ml/kg)	Inhibiting free-radical formation, restoration of the antioxidant systems (108).
			In vivo, rat TQ: (50 mg/L in drinking water) for 8 consecutive days.	Decreasing oxidative stress and preserving the activity of the anti-oxidant enzymes, ability to prevent the energy decline in kidney tissues (107).
		Ototoxicity	In vivo, rat TQ: (20 mg/kg, i.p.)	(130)
Antibiotic-aminoglycoside	Amikacin	Nephrotoxicity	In vivo, rat NSO: (2×0.5 ml)	(105)
Glycopeptide antibiotic	Vancomycin	Nephrotoxicity	In vivo, rat TQ: (10 mg/kg, i.p. continued at 24 h intervals for 8 days)	TQ can interact with ROS as a general radical scavenger (111).
Antibacterial (antimycobacterial)	Isoniazid	Hepatotoxicity	In vivo, rat *N. sativa* seeds powder: (1 g/kg/day, orally)	Antioxidant, anti-inflammatory (possibly through prevention of hydrazine function, one of INH metabolites), and anti-angiogenesis properties (54).
Antiretroviral drugs	Lamivudine, Zidovudine Efavirenz	Hepatotoxicity	In vivo, rat Aqueous extract of *N. sativa* seed (100, 200, 400 and 800 mg/kg, orally for 28 consecutive days)	(104)
NSAID	Acetaminophen	Nephrotoxicity	In vivo, rat TQ: (10 mg/kg orally)	Protection against lipid peroxidation and NO production (126).
			In vivo, mouse *N. sativa* aqueous extract: (300 mg/kg)	(127)
		Hepatotoxicity	In vivo, rat TQ: (2 mg/kg/day) for 5 days	Anti-oxidative and nitrosative stress and improving energy production in mitochondria, substantial increase in lowered ATP and GSH levels (98).
			In vivo, rat Alcoholic extract of *N. sativa*: (250 or 500 mg/kg, orally, for 7 days)	(100)
			In vivo, rat Three doses of TQ at a 15mg/kg total dose in an 18-htime interval	Upregulation of antioxidant systems (126)
NSAID	Indomethacin	Hepatotoxicity	In vivo, rat TQ: (10 mg/kg/day, orally, for 4 weeks)	Antioxidant and anti-apoptotic properties (102)
Anti-cancer Intercalating agent, antracyclines	Doxorubicin	Nephrotoxicity	In vivo, rat TQ (50 mg/kg/day, orally) for 3 weeks	Attenuating the oxidative stress, reversing the redox imbalance and subsequently ameliorating inflammatory tissue damage, Antioxidant activity through increasing Nrf2 expression and binding activity in renal tissue of DOX-treated rats (112).
			In vivo, rat TQ: (10 mg/kg per day, for 5 days)	(113)
		Cardiotoxicity	In vivo, rat TQ: (5days, 10 mg kg^-1^ day^-1^, p.o.)	Superoxide scavenging and anti-lipid peroxidation (30)
			In vivo, mouse TQ: (8 mg/kg/day, p.o.) for 5 days	(148)
Anticancer alkylating agents platinum compounds	Cisplatin	Hepatotoxicity	In vivo, rat Orally pre-treated with TQ: (500 mg. kg−1. day−1) for one month	Antioxidant properties and radical scavenging, prevention of inflammation through inhibition of inflammatory mediators like NO, TNF-α and IL-1β and suppression of NF-κB (62).
		Nephrotoxicity	In vivo, rat Alcoholic extract of NS (100 mg/kg)	(115)
			In vivo, rat TQ: (10 mg/kg b.w. in drinking water for 5 days)	Increasing anion and cation transporters and decreasing efflux transporters MRP2 and MRP4expression (114).
		Hematotoxicity	In vivo, mouse *N. sativa* aqua extract: (50 mg/kg, i.p.)	(157)
		Reproductive toxicity	In vivo, rat NSO: (0.5. ml/kg concomitantly with cisplatin)	(138)
Anti-cancer Alkylating agent	Cyclophosphamide	Cardiotoxicity	In vivo, rat TQ (50 mg/L in drinking water) for 5 days	Decrease oxidative and nitrosative stress, preserve the activity of antioxidant enzymes, improve the mitochondrial function and energy production (147)
		Hepatotoxicity	In vivo, rat NSO: (1ml/kg) TQ: (10mg/kg) Both via intragastric injection for 12 days	Antioxidant properties (69)
		Reproductive toxicity	In vivo, mouse Ethanol extraction of *N. sativa*: (10 mg/kg, i.p.)	(141)
Anticancer estrogen antagonist	Tamoxifen	Hepatotoxicity	In vivo, rat TQ: (50 mg/kg/day-,orally, for 20 consecutive days, starting 10 days before tamoxifen injection	Preventing oxidative stress and lipid peroxidation, enhancing antioxidant enzymes activities and inhibiting hepatic inflammation (76)
Anticancer Antimetabolite, DHFR inhibitor	Methotrexate	Hepatorenal toxicity	In vivo, rat TQ: (10mg/kg/day by gastric gavage for 10 consecutive days)	Antioxidant, antinitrosative, anti-inflammatory, and antiapoptotic mechanisms (66)
		Hepatotoxicity	human (Egyptian Children) Black seeds: (80 mg/kg/day, orally for one week after each methotrexate dose)	(65)
		Reproductive toxicity	In vivo, mouse TQ: (10 mg/kg/day, i.p. for 4 days)	(137)
		Gastrointestinal toxicity	In vivo, rat NSO: (10 ml/kg)	(155)
Anticancer Alkylating agent	Ifosfamide	Nephrotoxicity	In vivo, mouse TQ: (10 mg/kg per day, orally)	(119)
Antiepileptic	Sodium valproate	Hepatotoxicity	In vivo, rat TQ: (5-5.5mg/kg/day, in drinking water, for 21 days)	Antioxidant properties and cleansing the free radicals (101)
Immunosupressive	Cyclosporine	Nephrotoxicity	In vivo, rat NSO: (2 ml/kg orally)	(123)
		Cardiotoxicity	In vivo, rat NSO: (21 days, 2 ml/kg, orally)	Protect against oxidative stress and imbalance between the production of reactive oxygen species and endogenous antioxidant defense systems (151)
Gout drug alkaloyid	Colchicine	Reproductive toxicity	In vivo, rat Aqueous suspension of powdered *N.sativa* seeds: (1000 mg/kg/day)	(146)
Anticancer Glycopeptide antibiotic	Bleomycin	Pulmonary toxicity	In vivo, rat TQ: (5 mg/100mL, orally)	Superoxide scavenging and anti-lipid peroxidation (161)
